# Superficial Mastoid Abscess: Its Significance and Treatment

**Published:** 1907-05-25

**Authors:** 


### Otology.

### SUPERFICIAL MASTOID ABSCESS:

### Its Significance and Treatment.

In the great majority of cases superficial mastoid abscess is due to middle-ear disease, and demands, of course, other measures than mere incision and drainage. Very occasionally a superficial mastoid suppuration is due to furunculosis of the meatus or to a septic condition of the scalp or of the pinna. The following is an instructive case of this latter, not very common, class, the recognition of which is of primary importance in determining operative treatment. A young child was seen with purulent otorrhœa and an abscess over the right mastoid. The abscess was opened by simple incision, but the child did not do well. Preparations were made for opening the mastoid cells, as it was thought, quite properly, that the superficial abscess denoted disease within the bone. An examination of the drum-head had not, however, been successful, but the case appeared to be so clearly one of the class more usually met with that the practitioner, who had not doubted his own diagnosis, had arranged for operation by an aural surgeon. As soon as the child was anæsthetised the surgeon proceeded to examine the ear. He observed, however, over the temporal region two small dusky areas, quite recent scars of what might have been a little superficial ulceration. Bearing in mind that the tympanic membrane had not been seen, the meatus was cleansed, and the membrane was examined, and found to be perfectly normal. There was no injection of the blood-vessels, no perforation, and all the landmarks could be clearly seen. On the strength of these observations it was concluded that the mastoid abscess was due not to middle-ear disease, but to lymphatic absorption from impetigo. On enlarging the existing wound, it was evident that the otorrhœa was due to rupture of the abscess into the external auditory meatus. The abscess cavity over the mastoid was laid open more freely and thoroughly drained, the periosteum was left intact, and the ear saved.

On the other hand, the following case illustrates the fatal danger of treating superficial mastoid abscess by simple incision alone when due to middle-ear disease, and also of postponing operation on the mastoid or tympanum until the onset of brain symptoms emphasises the danger of delay. A child under one year old had had superficial mastoid abscess and otorrhœa. The abscess had been opened and drained, and though for a time the child improved, the wound did not completely heal. The child during the next two months was ill with pyrexia and diarrhœa, but it was not realised, owing to the absence of external signs, other than otorrhœa and mastoid sinus, that these general symptoms were caused by the local disease. Not until head symptoms suddenly set in was the sinus opened up and bare bone found: a portion of the necrosed mastoid was removed, but the child succumbed to the meningitis which had arisen. The post-mortem revealed that the temporal bone had been the seat of chronic infective disease, with necrosis, which had set up recent meningitis over the cerebrum.

Those cases of otorrhœa which persist after incision and drainage of a superficial mastoid abscess should also be considered from the point of view of mastoid operation, even without the existence of a mastoid sinus. Merely to make an incision down to the mastoid, in the case of superficial mastoid abscess associated with middle-ear disease, is almost always useless unless followed up by draining of the mastoid cavity. The simple incision is, moreover, except as a tentative measure, actually dangerous, for it gives a sense of false security to patient and doctor alike.

